# Hedgehog-GLI mediated control of renal formation and malformation

**DOI:** 10.3389/fneph.2023.1176347

**Published:** 2023-04-20

**Authors:** Dina Greenberg, Robert D’Cruz, Jon L. Lacanlale, Christopher J. Rowan, Norman D. Rosenblum

**Affiliations:** ^1^ Program in Developmental and Stem Cell Biology, Hospital for Sick Children, Toronto, ON, Canada; ^2^ Department of Laboratory Medicine and Pathobiology, University of Toronto, Toronto, ON, Canada; ^3^ Division of Nephrology, Hospital for Sick Children, Toronto, ON, Canada; ^4^ Department of Pediatrics, University of Toronto, Toronto, ON, Canada

**Keywords:** kidney, CAKUT, hedgehog signaling, stromal, ureteric, nephrogenic, Pallister-Hall syndrome

## Abstract

CAKUT is the leading cause of end-stage kidney disease in children and comprises a broad spectrum of phenotypic abnormalities in kidney and ureter development. Molecular mechanisms underlying the pathogenesis of CAKUT have been elucidated in genetic models, predominantly in the mouse, a paradigm for human renal development. Hedgehog (Hh) signaling is critical to normal embryogenesis, including kidney development. Hh signaling mediates the physiological development of the ureter and stroma and has adverse pathophysiological effects on the metanephric mesenchyme, ureteric, and nephrogenic lineages. Further, disruption of Hh signaling is causative of numerous human developmental disorders associated with renal malformation; Pallister-Hall Syndrome (PHS) is characterized by a diverse spectrum of malformations including CAKUT and caused by truncating variants in the middle-third of the Hh signaling effector GLI3. Here, we outline the roles of Hh signaling in regulating murine kidney development, and review human variants in Hh signaling genes in patients with renal malformation.

## Introduction

Congenital Anomalies of the Kidney and Urinary Tract (CAKUT) constitute 20% of all congenital malformations and are the leading cause of end-stage kidney disease in children (1, 2). CAKUT comprises a broad phenotypic spectrum consisting of a range of kidney (renal agenesis, renal aplasia, renal dysplasia, hydronephrosis, multicystic dysplastic kidney, ectopic kidney, horseshoe kidney, renal tubular dysgenesis) and ureter (ureteropelvic junction obstruction (UPJO), vesicoureteral reflux (VUR), duplex collecting system, megaureter) anomalies (3, 4). While some forms of CAKUT can be mild with little or no functional impact, severe CAKUT at birth is associated with greater risk for chronic kidney disease and kidney replacement therapy (KRT), as well as increased risk of death in preterm infants (5, 6). CAKUT can appear as an isolated condition, and less commonly in syndromic form in concert with other extrarenal manifestations (i.e., defects in other organ systems) (7).

CAKUT is caused by disruption of the processes that regulate renal morphogenesis; this can be a result of *in utero* environmental, epigenetic, or genetic factors (3). To date, most investigations have focused on monogenic causes of CAKUT, with causative genes identified in both isolated and syndromic forms. However, monogenic CAKUT only accounts for 15-20% of all CAKUT cases, and poses a diagnostic challenge owing to incomplete penetrance and variable phenotypic expression (8–11). Other genetic causes of CAKUT include copy number variations (CNVs), chromosomal abnormalities, and, less commonly, oligogenic or polygenic CAKUT (7, 12–16).

Understanding the process of renal formation is key to elucidating the mechanisms which give rise to CAKUT. Human kidney development begins at week 3 of embryonic development with the formation of the pronephros, which regresses and is followed by formation of the mesonephros at 4 weeks and the metanephros at 5 weeks gestational age. Murine kidney development begins with reciprocal inductive interactions between the nephric duct (ND) and the metanephric mesenchyme (MM), which originate at embryonic day 8 (E8.0) from the anterior and posterior *Osr1*-expressing intermediate mesoderm (IM), respectively (17–19). At E10.5, inductive signaling from the MM stimulates the outgrowth of the ureteric bud (UB) from the ND. In the developing human embryo, this correlates with metanephros development at 5 weeks gestational age (20). The UB bifurcates into a T-shaped structure at E11.5 and undergoes iterative rounds of branching morphogenesis up until post-natal day 2 (P2) (or 34 weeks of human gestation); a process that ultimately gives rise to the mature renal collecting system (21–24). In parallel, inductive signaling from the UB signals the MM to differentiate into two distinct multipotent self-renewing progenitor populations: the *Foxd1*-expressing stromal and *Six2* -expressing nephron progenitor lineages (18, 25, 26). The *Foxd1*-expressing stromal lineage subsequently gives rise to endothelial cells, vascular smooth muscle cells, pericytes, mesangial cells, and fibroblasts (25, 27). *Six2*-expressing nephron progenitors undergo mesenchymal-to-epithelial transition (MET) to form renal vesicles, wherein proximal/distal polarity is established. Polarized renal vesicles subsequently develop into comma-shaped bodies, S-shaped bodies, and finally, into mature nephrons (28). Murine nephrogenesis continues up until P4, while human embryonic nephrogenesis terminates at week 36 of gestation (29, 30). Overall, these processes are tightly regulated by a number of signaling pathways and factors, disruption of which has been shown to contribute to the phenotypic spectrum of CAKUT.

## Hedgehog signaling

Hedgehog (Hh) signaling controls mammalian organogenesis, including that of the kidney (31). Dysregulation of Hh signaling is associated with various types of cancers, such as medullablastoma and basal cell carcinoma (32, 33). Activation of the Hh pathway can occur through a canonical (Glioma-Associated Oncogene (GLI)-dependent) or non-canonical (GLI-independent) mechanism. While non-canonical activation of the Hh pathway has been implicated in cancer, its role in mammalian kidney development has not yet been interrogated (34–36). Mammalian canonical Hh signaling is initiated when Hh ligand binds and inactivates the transmembrane protein receptor Patched1 (PTCH1), which results in PTCH1 being internalized and degraded. Degradation of PTCH1 results in accumulation of Smoothened (SMO), an intracellular receptor, at the primary cilium and activation of downstream cascades resulting in the processing and translocation of GLI transcription factors into the nucleus (32, 37). Absence of Hh ligand causes PTCH1 binding and inhibition of SMO activity. This results in phosphorylation of downstream GLI proteins, which are then targeted for processing. Phosphorylation of GLI proteins by combinations of Protein Kinase A and C (PKA/C), Casein Kinase 1 (CK1), Glycogen Synthase Kinase 3β (GSK3β), and Dual-Specificity Yak1-Related Kinase (DYRK1) targets GLI proteins for proteasome-dependent processing, giving rise to a truncated repressor form that translocates to the nucleus to repress Hh target gene transcription. In vertebrates, there are three GLI family members: GLI1, GLI2, and GLI3. GLI1 functions as a transcriptional activator, while GLI2 and GLI3 can function as both activators or repressors depending on their truncation. The major determinant of a cellular response to Hh ligand is the ratio of GLI activator to GLI repressor (GLIA/GLIR), as this ratio has been shown to control processes of organogenesis (38, 39).

## Hedgehog signaling in kidney development

In mammals, there are three Hh genes, Sonic Hedgehog (Shh), Indian Hedgehog (Ihh), and Desert Hedgehog (Dhh). Of these, only Shh and Ihh expression is detectable in the developing mammalian kidney; Shh is expressed in the ureteric lineage and localizes to the epithelium of the presumptive ureter and medullary collecting ducts, while Ihh is localized to the nephrogenic tubules (40–43).

**Figure 1:**
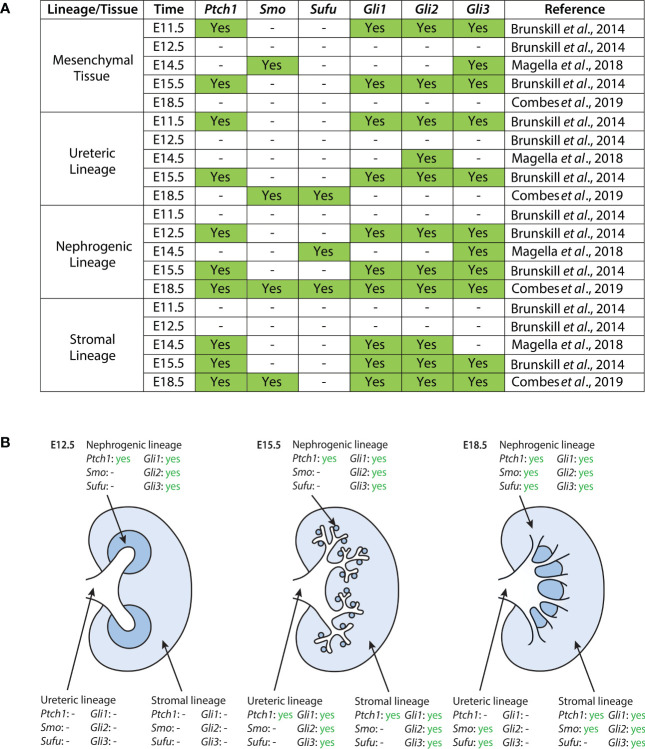
Expression of Hh components in the developing mouse kidney as reported in published single-cell RNA sequencing datasets (40, 41, 46). **(A)** Datasets were screened for expression of *Ptch1*, *Smo*, *Sufu*, *Gli1*, *Gli2*, and *Gli3*. Expression here is reported as binary; “yes” indicates statistically significant expression of a gene, as determined by the study. The exception to this is the dataset from Magella et al. (E14.5), which reports a cluster of greatest correlation for each gene; here, we include correlations based on both the Fluidigm and Chromium platforms, as they are the ones used by Brunskill et al. and Combes et al., respectively. A dash indicates either no statistically significant expression of a gene within a lineage cluster, or the absence of expression data reported altogether. **(B)** Expression of Hh pathway components in the developing mouse kidney at E12.5, E15.5, and E18.5. Data shown here is provided in greater detail in **(A)**.

Previous studies have shown that the GLI family of transcription factors control the expression of cell cycle regulators *N-myc* and *CyclinD1* (44), as well as genes critical to renal patterning such as *Foxd1*, *Pax2*, *Sall1*, and *Tgfb2* (44, 45). Studies in multiple organ systems and cancers have also demonstrated that GLI transcription factors activate other Hh pathway components such as *Ptch1*, *Smo*, *Gli1*, *Gli2*, and *Gli3*, leading to positive and negative feedback of the Hh signaling pathway. Analysis of the temporospatial localization of these Hh signaling factors has therefore provided evidence of important roles for Hh signaling across murine and human kidney development. These studies are outlined below and are further supported by data drawn from published single-cell datasets of the developing mouse kidney (**Figure 1**).

### Ptch1

During murine kidney development, *Ptch1* expression is weakly localized to the epithelium of the presumptive ureter and medullary collecting ducts, and strongly expressed in the surrounding stromal mesenchyme (47). In the adult mouse kidney, *Ptch1* expression is localized at the corticomedullary junction (42). Single-cell data suggests that between E11.5 and E18.5, *Ptch1* is most strongly expressed in the mesenchymal and stromal clusters, with additional expression in the nephrogenic and ureteric clusters (40, 41, 46).

### Smo

Single-cell data suggests that in the E14.5 mouse kidney, *Smo* is expressed most strongly in the mesenchymal and endothelial cell lineages (46). Further, Combes et al. detected expression of *Smo* in the stromal, nephrogenic, and endothelial lineages of the E18.5 murine kidney (41).

### Gli1

Expression of Gli1 in the murine embryonic kidney is localized to the interstitial stromal cells (42, 44, 45). Single-cell data shown by Brunskill et al. further demonstrated low levels of *Gli1* expression in the ureteric lineage at E11.5 and E15.5, and *Gli1* expression in developing mesenchymal and stromal lineages (40, 41, 46).

### Gli2

*Gli2* is highly expressed within the medulla of the adult murine kidney but localized to the cortex at E14.5 (42, 44). Single-cell expression data suggests *Gli2* is largely expressed in the mesenchymal and stromal lineages, with additional expression in the nephrogenic and ureteric lineages (40, 41, 46).

### Gli3

Gli3 protein expression is ubiquitous at E14.5 (44). Single-cell expression data demonstrates that *Gli3* is highly expressed in mesenchymal clusters at E11.5 and E14.5, with subsequent expression in stromal and nephrogenic clusters. *Gli3* expression is also detected in the ureteric lineage at E11.5 and E15.5 (40, 41, 46).

## Physiological roles for hedgehog signaling in murine kidney development

**Figure 2:**
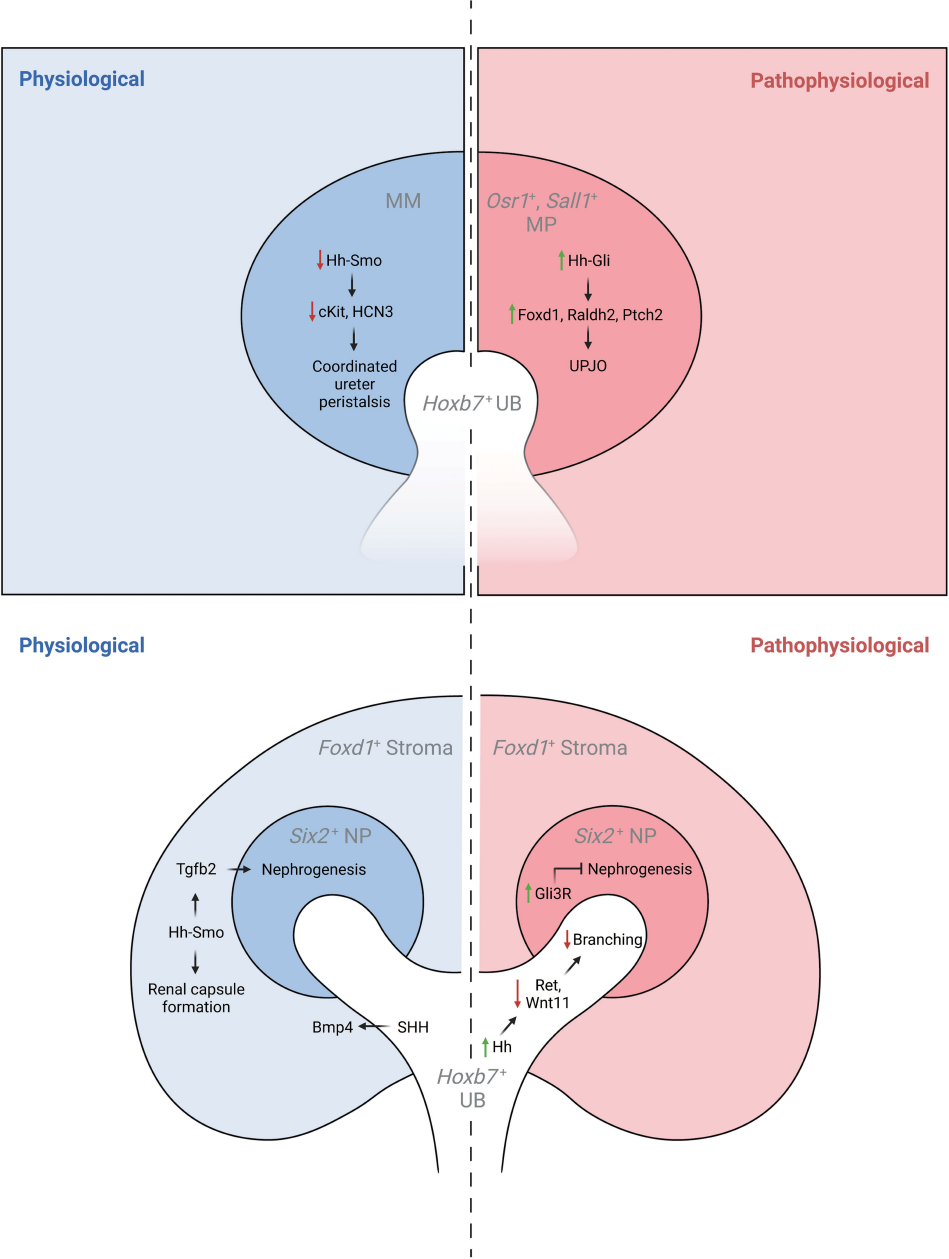
The physiological and pathophysiological roles of Hh signaling in specific lineages of the developing kidney. MM, metanephric mesenchyme; MP, mesenchymal progenitors; UB, ureteric bud.

Previous studies using genetic mouse models have demonstrated important physiological roles for Hh signaling during murine kidney development. Mice with global deficiency of *Shh* ligand display a spectrum of renal abnormalities including bilateral renal aplasia and ectopic dysplastic kidneys (44, 48). Subsequent studies have identified key physiological roles for Hh signaling in controlling ureter and stromal development. These studies are outlined below and summarized in **Table 1** and **Figure 2**.

**Table 1:** A summary of the mouse models used to investigate the physiological and pathophysiological roles of Hedgehog signaling in the developing kidney along with the associated phenotypes.

Model	Renal Phenotype	Study
*Hoxb7Cre;Shh^loxP/-^*	Hydroureter, hydronephrosis, reduced periureteric mesenchyme cell proliferation, delayed ureteral smooth muscle differentiation	Yu et al., 2002 (43)
*Rarb2Cre;Smo^loxP/-^*	Non-obstructive hydronephrosis and hydroureter, abnormal ureter peristalsis	Cain et al., 2011 (49)
*Foxd1Cre;Smo^loxP/-^*	Defects in cortical stromal patterning (abnormal capsular morphology), loss of cortical stromal markers, increased stromal cell apoptosis, renal hypoplasia, expanded nephron progenitor domain and reduced nephron number	Rowan et al., 2018 (45)
*Foxd1Cre;Gli3^Tflag/+^;Gli3^Δ699/+^*	Phenocopies *Foxd1Cre;Smo^loxP/-^*	Rowan et al., 2018 (45)
*Rarb2Cre;Ptch1^loxP/-^*	Obstructive hydronephrosis due to ectopically localized stromal cells occluding the ureteropelvic region	Sheybani-Deloui et al., 2018 (50)
*Sall1Cre;Ptch1^loxP/-^*	Same as *Rarb2Cre;Ptch1^loxP/-^*	Sheybani-Deloui et al., 2018 (50)
*Osr1Cre;Ptch1^loxP/-^*	Same as *Rarb2Cre;Ptch1^loxP/-^*	Sheybani-Deloui et al., 2018 (50)
*Hoxb7Cre;Ptch1^loxP/-^*	Ectopic Hh signaling, impaired ureteric cell-specific gene expression, renal hypoplasia, sparse mature and intermediate nephrogenic-derived structures	Cain et al., 2009 (47)
*Gli3 ^Δ699/Δ699^*	Hydroureter, hydronephrosis, thin renal cortex, renal hypoplasia	Blake et al., 2016 (51)
*Six2Cre;Rosa26^Gli3TFLAG/+^*	Reduced nephron intermediate structures, *Six2^+^* nephron progenitors, abnormal patterning of nephron progenitors, and renal hypoplasia	Blake et al., 2016 (51)

Models exploring the physiological roles are denoted in blue and pathophysiological in red.

### Ureter development

Genetic inactivation of *Shh* in the ureteric lineage results in hydroureter and hydronephrosis, a reduction in periureteric mesenchyme cell proliferation, and delayed ureteral smooth muscle differentiation associated with reduced expression of *Bmp4* and *Ptch1* (43). Further, targeted inactivation of *Smo* in intermediate mesenchyme that gives rise to the metanephros (*Rarb2Cre;Smo^lox/P-^*) results in non-obstructive hydronephrosis and hydroureter. In contrast to mice with genetic deficiency of *Shh* in the ureteric lineage, *Rarb2Cre;Smo^loxP/-^* mice exhibit normal ureteric smooth muscle but abnormal ureter peristalsis and a reduction in expression of urinary tract pacemaker cell (utPMC) markers *cKit* and *Hcn3* (49, 52). Together, these findings demonstrate a physiologic role for Hh signaling in regulating urinary tract pacemaker activity and development of urinary pacemaker cells.

### Stromal development

*Foxd1*+ stromal cells play important roles in regulating renal development (53). Expression data demonstrates that *Gli1* is present in the developing cortical stroma, suggesting a role for Hh signaling in cortical stromal function (42, 45) and single-cell RNA sequencing demonstrates expression of *Gli1* in stromal clusters starting at E14.5 (40, 41, 46). Recent studies have demonstrated that genetic deletion of *Smo* in the stromal lineage (*Foxd1Cre;Smo^loxP/-^*) causes loss of the cortical stroma after *Foxd1+* cells initially migrate to take up residence in the renal cortex, resulting in loss of cortical stromal markers such as *Foxd1* and *Raldh2*, and near-absence of the renal capsule (Rowan, 2018). Inactivation of stromal Hh signaling also negatively regulates the nephrogenic domain, as *Foxd1Cre;Smo^loxP/-^* mice exhibit renal hypoplasia with an expanded nephron progenitor domain and fewer nephrons (45). The contribution of GLI3R to these defects was demonstrated in mice with obligate GLI3R expression targeted to the stroma (*Foxd1Cre;Gli3^Tflag/+^;Gli3^Δ699/+^*), in which these defects were replicated, and in mice with genetic deletion of *Gli3* in a *Foxd1Cre;Smo^loxP/-^* background, in which these abnormalities were rescued (45). While the downstream mediators of these Hh-dependent effects are only beginning to be elucidated, it has been demonstrated that *Tgfb* signaling acts downstream of Hh signaling to exert cell autonomous and non-cell autonomous functions (45).

## Pathophysiological roles for hedgehog signaling in murine kidney development

Increased Hh activity in non-renal tissue causes cancer of different types and has been shown to be pathogenic during lung and limb development (54–56). Here, we review how Hh signaling targeted to specific embryonic kidney cell lineages is detrimental to renal morphogenesis. These studies are reviewed below and summarized in **Table 1** and **Figure 2**.

### Metanephric mesenchyme

Sheybani-Deloui et al. investigated the role of Hh constitutive activation in cells giving rise to the metanephric mesenchyme using genetic mouse models with Cre-mediated excision of *Ptch1.* Tamoxifen-induced Cre-mediated *Ptch1* deficiency in *Osr1+* cells, the progenitors to both nephrogenic and stromal cells, starting at E9.5 caused obstructive hydronephrosis and ureteropelvic junction (UPJ) obstruction in embryonic kidneys. Investigation of underlying cellular and molecular mechanisms demonstrated ectopic localization of *Foxd1+* and *Raldh2*+ cells at the UPJ with resulting blockage of urine outflow. Lineage tracing experiments confirmed that these blocking cells are, indeed, derived from metanephric mesenchyme. Analysis of RNA expressed by these cells revealed other markers of the cortical stroma and Hh-targets. Analysis of UPJ tissue in children with UPJO demonstrated expression of both stromal and Hh-target genes in a subset of affected patients (50). Together, these findings suggest that increased Hh signaling in *Osr1+* progenitor cells during a narrow time window causes abnormal stromal cell localization to the UPJ with resultant obstruction.

### Ureteric lineage

Increased Hh signaling activity in the *Hoxb7+* ureteric lineage via deletion of *Ptch1* (*Hoxb7Cre;Ptch1^loxP/-^*) results in ectopic Hh signaling in ureteric branch tips, impaired expression of genes specific to ureteric tip cells, and renal hypoplasia. Overexpression of GLI3R in mice with *Ptch1-*deficiency in the ureteric lineage rescued the normal pattern of Hh signaling and expression of ureteric genes as well as renal hypoplasia (47).

Germline expression of GLI3R (*Gli3^D699/D699^*) results in a complex CAKUT phenotype consisting of hydroureter, hydronephrosis, a thin renal cortex, and renal hypoplasia. Among the mechanisms underlying these phenotypes is impairment of ureteric bud lengthening (51). Collectively, these studies demonstrate that regulation of Hh activity and GLI3R expression is critical to development of the ureteric bud and its branches.

### Nephrogenic lineage

Obligate expression of GLI3R (*Six2Cre;Rosa26^Gli3TFLAG/+^*) targeted to the *Six2^+^* nephrogenic lineage inhibits nephrogenesis via inhibition of *Six2+* progenitor cell self-renewal resulting in renal hypoplasia (51).

## Hh signaling in human kidney development

Below, we outline human renal malformations characterized by disruption of genes required for Hh signaling.

### SHH

**Figure 3:**
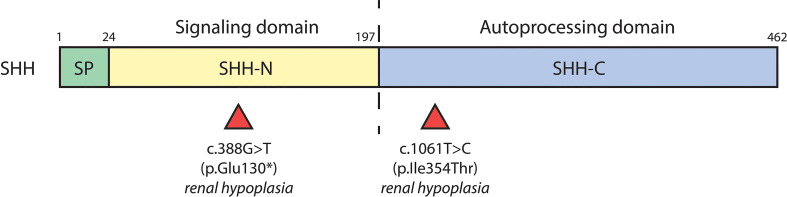
*SHH* variants associated with CAKUT. Schematic diagram of the biochemical domains of human SHH shows the signal peptide (SP, a.a. 1-23), signaling domain (SHH-N, a.a. 24-197), and autoprocessing domain (SHH-C, a.a. 198-462). The dashed line indicates the site of autocatalytic cleavage. Red triangles underneath denote location of *SHH* variants and their associated renal phenotypes.

Numerous cases of CAKUT in patients carrying terminal deletions in the long arm of chromosome 7q32-36 (encompassing the *SHH* locus) have been reported with phenotypes including renal hypoplasia, renal dysplasia, ectopic kidney, duplex collecting system and VUR (**Table 2**). Patients had other extrarenal manifestations including, but not limited to, holoprosencephaly, facial dimorphisms, and developmental delay (57–60). Several *SHH* variants have further been identified as resulting in CAKUT; a nonsense mutation (c.388G>T) resulting in truncation at exon 2 of SHH caused renal hypoplasia along with holoprosencephaly and facial dimorphisms in a male patient (61). A female patient carrying the c.1061T>C missense mutation in exon 3 of *SHH* had renal hypoplasia, holoprosencephaly, cerebellar, and retinal defects (61). *SHH* encompasses 3 exons; nascent SHH undergoes proteolysis to generate an N-terminal (N-SHH; comprising of exons 1 and 2) or C-terminal (C-SHH; comprising of exons 1 and 3) form (**Figure 3**). Although it is N-SHH that ultimately binds PTCH1 to initiate the Hh signaling cascade, C-SHH is critical to the processing of N-SHH (62, 63). Thus, disruption to *SHH* may be detrimental to human renal morphogenesis.

**Table 2:** *SHH* and *GLI2* variants identified in humans and their associated renal phenotypes.

Affected gene	Variant	Mutation type	Renal phenotype	Study
*SHH*	c.388G>T	Truncation	Renal hypoplasia	Dubourg et al., 2004 (61)
*SHH*	c.1061T>C	Missense	Renal hypoplasia	Dubourg et al., 2004 (61)
*GLI2*	c.3369delG	Truncation	Renal hypoplasia (left kidney), cystic dysplasia (right kidney)	Shirakawa et al., 2018 (64)
*GLI2*	c.3332delT	Truncation	Renal hypoplasia, VUR	Babu et al., 2019 (65)
*GLI2*	c.1723T>C	Truncation	Bilateral renal hypoplasia	Babu et al., 2019 (65)

### GLI2

**Figure 4:**
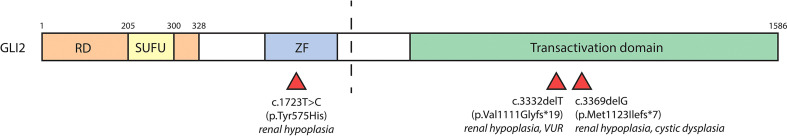
*GLI2* variants associated with CAKUT. The human GLI2 protein is comprised of a repressor domain (RD, a.a. 1-328), SUFU-binding domain (SUFU, a.a. 205-300), zinc finger DNA-binding domain (ZF) and C-terminal transactivation domain. The zinc finger and transactivation domains of GLI2 have not been well positionally defined. The dashed line indicates the site of proteolytic cleavage that results in formation of GLI2R. Red triangles underneath denote location of *GLI2* variants and their associated renal phenotypes.

*GLI2* variants have been linked to polydactyly and dysmorphic facial features. There have only been three recorded cases of *GLI2* variants resulting in CAKUT, outlined in **Table 2** and **Figure 4**. One study identified a male carrying one copy of a c.3369delG *GLI2* allele, which was predicted to generate a truncation in the C-terminal GLI2 transactivation domain. The patient presented with a left hypoplastic kidney, a right cystic dysplastic kidney, as well as urinary tract obstruction, eventually progressing to end-stage-renal failure. Other features included postaxial polydactyly and cognitive developmental delay (64). Another study identified a female with a heterozygous c.3332delT *GLI2* variant, similarly predicted to cause C-terminal truncation. The patient had renal hypoplasia and VUR, as well as congenital heart disease, cleft palate, deafness, ocular, and cognitive impairment. The same study identified another female patient with a heterozygous c.1723T>C *GLI2* variant with bilateral renal hypoplasia, polydactyly, cleft palate, craniofacial abnormalities, and pituitary hypoplasia. The c.1723C>T variant was predicted to cause truncation in the C-terminal portion of the DNA binding domain of GLI2. *In vitro* co-transfection of a truncated c.1723C>T *GLI2* plasmid with a full length GLI2 plasmid showed a 55% reduction in transcriptional activation (65). GLI2 is regarded as a potent activator of Hh signaling, but owing to inefficient processing, is a weak transcriptional repressor, suggesting a mechanism of loss of function of GLI2A that results in decreased Hh activity, rather than constitutive repression (66, 67).

### GLI3

Human variants in the *GLI3* gene are known to cause Pallister-Hall Syndrome (PHS; OMIM: 146510) and Greig cephalopolysyndactyly syndrome (GCPS; OMIM: 175700), and non-syndromic postaxial polydactyly types A1 and B (PAPA1/PAPB; OMIM: 174200) and preaxial polydactyly type IV (PPD-IV; OMIM: 174700). Congenital diseases driven by mutations in *GLI3* are inherited in an autosomal dominant fashion and give rise to a broad spectrum of clinical phenotypes. A diagnosis of Pallister-Hall Syndrome is met upon the criteria of hypothalamic hamartoma, mesoaxial polydactyly, and a confirmed mutation in the central-third of the *GLI3* gene (68–71). PHS is characterized by a further spectrum of multi-organ malformations, including CAKUT (71, 72). An estimated 27% of PHS patients present with CAKUT, with hypoplasia (with or without dysplasia) and agenesis being most common (accounting for just under one third of PHS cases with CAKUT, each). Other types of CAKUT found in PHS include dysplasia, ectopic kidney, single kidney, and VUR, all of which are reported to occur unilaterally and bilaterally at equal frequency. PHS patients with CAKUT are more likely to experience craniofacial defects, bifid epiglottis, and genital hypoplasia (72).

Several large patient cohort studies have allowed for the identification of distinct genotype-phenotype correlations of *GLI3* variants (71–76). Patients with PHS have truncating variants (frameshift or nonsense) almost exclusively within the central-third (amino acids 661-1159) of the *GLI3* gene. As these truncations take place C-terminally to the zinc finger DNA-binding domain of GLI3 and cause loss of the N-terminal transactivation domain, it is believed that the resulting protein mimics a constitutively active GLI3R (74, 77, 78). It has been proposed that constitutive GLI3R activity is the driver of CAKUT phenotypes in PHS; this notion is supported by the exclusive association of CAKUT with PHS-causing *GLI3* variants, as well as the requirement for balanced GLI3A and GLI3R activities in normal kidney formation (31, 44, 45, 51, 79). Indeed, *GLI3* variants found in GCPS or isolated polydactylies, which have no renal manifestations, are proposed to cause functional haploinsufficiency of the GLI3 protein (74–76).

Although all PHS-causing *GLI3* variants are predicted to generate GLI3R-like transcripts, only a quarter of PHS patients present with CAKUT. A recent patient cohort study by McClelland et al. investigated the link between *GLI3* variants and renal phenotype in PHS patients; the study demonstrated that PHS-causing *GLI3* variants are evenly distributed within the central third region of the *GLI3* gene, and found no correlation between locus of mutagenesis and prevalence of CAKUT (72). It may be, however, that the spectrum of renal phenotypes found in PHS patients is mediated by *GLI3* variant-specific mechanisms that are difficult to elucidate from genotype alone.

*GLI3* mRNA stability may be an important mediator of *GLI3* variant induced renal phenotype. One study identified three cases of *GLI3* variants (c.1320_1321insT, c.2372delC, and c.2374C>T) in patients with isolated polydactyly that were subject to nonsense-mediated mRNA decay (NMD). C.2372delC and c.2374C>T fall within the central region of *GLI3* that is typically associated with PHS; interestingly, the c.2374C>T variant has been identified in six other cases of GCPS (74, 80). Given that GCPS is characterized by loss of GLI3 function, whereas PHS is believed to arise from constitutive GLI3R activity, it is possible that PHS-causing *GLI3* variants are less likely be subject to NMD. Further investigation is required to elucidate the contribution of NMD to CAKUT within the PHS phenotypic spectrum.

**Figure 5:**
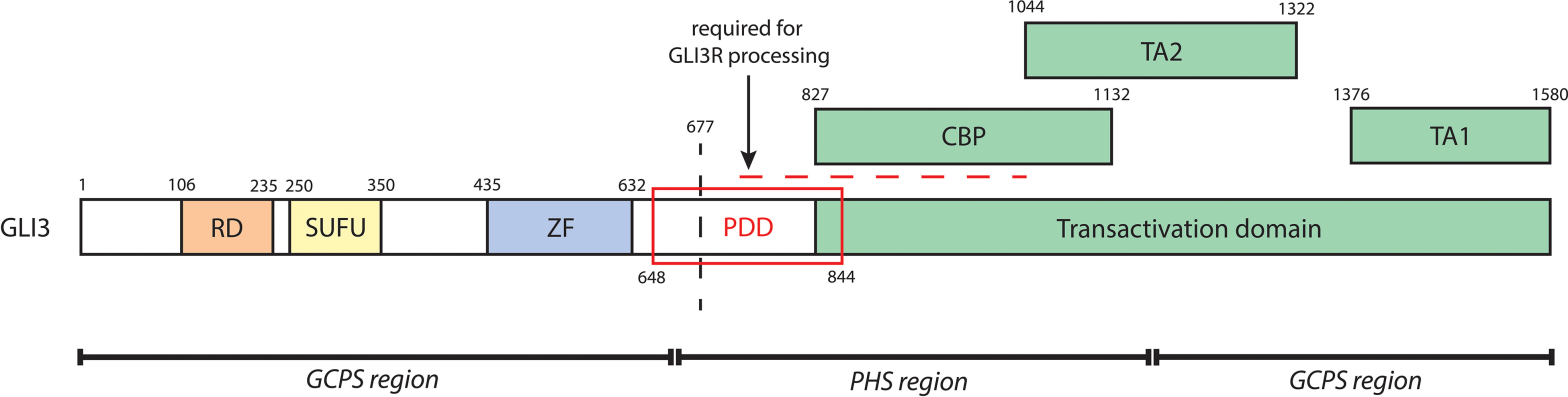
A schematic of biochemical domains of human GLI3 containing the repressor domain (RD, a.a. 106-235), SUFU-binding domain (SUFU, 250-350), zinc finger DNA-binding domain (ZF, a.a. 435-632), and transactivation domain (a.a. 827-1580). The transactivation domain, which is critical to GLI3A, contains a CREB-binding protein domain (CBP, a.a. 827-1132), transactivation domain 2 (TA2, a.a. 1044-1322), and transactivation domain 1 (TA1, a.a. 1376-1580). The processing determinant domain (PDD, a.a. 648-844) is critical to the proteolytic cleavage of GLI3. The horizontal dashed line further denotes a series of sites required for GLI3R processing; GLI3 is phosphorylated at its PKA sites (a.a. 849, 869, 877, 907, 980, 1006) and GSK3β sites (a.a. 861, 873, 903). This leads to binding of βTrCP (at GLI3 a.a. 856, 864, 855, 894) and subsequent ubiquitination by SCF^TrCP^ (at GLI3 a.a. 773, 779, 784, 800), causing proteolytic cleavage (indicated by the vertical dashed line) and thus generation of GLI3R. Regions of mutagenesis of *GLI3* associated with either GCPS (a.a. 1-660, 1160-1580) or PHS (a.a. 661-1159) are indicated at the bottom.

Renal phenotype in PHS may also be a consequence of GLI3 variant structure and function. Physiological processing of nascent GLI3 into GLI3A or GLI3R requires interaction with multiple binding partners at domains spanning the full length of GLI3, outlined in **Figure 5**. PHS-causing variants fall within the central region of GLI3 (amino acids 661-1159), and without exception truncate the TA1 and TA2 domains, thus disrupting GLI3A activity. It is possible that variant-specific positional truncation of GLI3 is determinant of the success of posttranslational modification, particularly within regions critical to GLI3R processing, and subsequent proteolytic cleavage (67, 81). Alternatively, it may be that truncated GLI3 variants are of sufficient structural similarity to wildtype proteosomally cleaved GLI3R and are thus able to emulate GLI3R activity. Future studies of patient *GLI3* variants would be beneficial to our understanding of the molecular mechanisms underlying phenotypic variation in PHS.

Lastly, given the extensive network of genes that govern kidney development, it is also possible that the variable penetrance of CAKUT in PHS may be a factor of not only specific *GLI3* variants, but also secondary genetic background factors (i.e., modifier gene interactions, stochastic effects, epigenetic mechanisms), as has been demonstrated in other human diseases (3, 82).

Further investigation is required to elucidate the molecular mechanism by which *GLI3* variants associated with PHS give rise to variable penetrance of CAKUT. While studies in mouse models have shed light on the effects of constitutive GLI3R activity on kidney development, the hemizygous *Gli3^Δ699/+^* mouse model is phenotypically normal, indicating that there are likely differences in Hh-mediated regulation between murine and human kidney development. For example, there may be variability in Hh sensitivity across human and mouse kidney cell lineages. Similarly, it appears that GLI3R exerts its effects on mouse and human tissue at different dosages. Use of a human kidney organoid model would permit the interrogation of these key differences. Kidney organoids could be used to assay the impacts of Hh signaling on distinct kidney cell lineages, as well as the effects of gain of function or loss of function of Hh signaling. Changes to Hh signaling and kidney development driven by specific *GLI3* variants can be similarly investigated in kidney organoids. Further, the use of an isogenic kidney organoid model, where possible, has the added benefit of preservation of genetic background, which may be an important contributor to the phenotypic variance seen in PHS, as well as other forms of CAKUT (83, 84).

## Conclusion

CAKUT represents a broad spectrum of kidney and urinary tract malformations that are a significant source of morbidity in the pediatric population. Dysregulation of genes associated with normal kidney and ureter development lead to the development of CAKUT. Previous work using genetic mouse models has demonstrated that Hh signaling plays important physiological and pathophysiological roles in kidney and ureter morphogenesis, and mutations in Hh genes have been shown to cause human renal malformation. Future studies using a combination of genetic mouse models and human kidney organoids can help elucidate the precise variant-specific molecular mechanism underlying Hh-mediated renal malformation.

## Author contributions

All authors contributed to conception and design of the review. DG, RD, and JL wrote the manuscript. All authors contributed to manuscript revision and approved the final manuscript. All authors contributed to the article and approved the submitted version.
